# The presence of a fat layer after neoadjuvant chemotherapy as an indicator of prognosis in osteosarcoma

**DOI:** 10.3389/fonc.2025.1514560

**Published:** 2025-04-11

**Authors:** Junqi Huang, Zengru Xie

**Affiliations:** Department of Orthopaedics, First Affiliated Hospital of Xinjiang Medical University, Urumqi, China

**Keywords:** MRI, osteosarcoma, chemotherapy, prognosis, survival

## Abstract

**Objective:**

This study aimed to evaluate the potential of magnetic resonance imaging (MRI) to monitor the response in patients with osteosarcoma receiving chemotherapy and to assess the correlation between the presence of a fat layer surrounding the tumor after neoadjuvant chemotherapy and prognosis.

**Methods:**

In total, 28 patients with osteosarcoma were included in this retrospective study. All patients underwent chemotherapy and surgery. MRI scans of the patients were evaluated before and after neoadjuvant chemotherapy. The prognostic factors included histological response and alkaline phosphatase (ALP) level. Relapse and survival at follow-up were defined as patient outcomes. The log-rank test was used to compare these factors with various MRI characteristics (e.g. change in maximum lesion length before and after chemotherapy, change in maximum edema, and fat layer presence after chemotherapy).

**Results:**

The median time of follow-up was 64.3 ± 41.5 months. The 3- and 5-year event-free survival rates were 75.0% and 67.9%, respectively. ALP levels after chemotherapy were associated with tumor necrosis (p = 0.01). Change in maximum lesion length [p = 0.044; odds ratio (OR) = 0.035; confidence interval (CI): 0.01–0.911] was a predictor of survival. Changes in edema on T2-weighted sequences (p = 0.979; OR = 0.989, CI: 0.437–2.242) were not significant. The presence of a fat layer (p = 0.013; OR = 0.000; confidence CI: 0.000–0.018) predicted good event-free survival.

**Conclusions:**

The presence of a fat layer correlated with good prognosis in patients with osteosarcoma. MRI characteristics in the early stages could help to inform decision-making about treatment strategy.

## 1 Introduction

Osteosarcoma is the most common primary malignant bone tumor in children and young adults, particularly during the second decade of life (1). The use of high-dose methotrexate before surgery was introduced in the 1970s to improve prognosis in patients with osteosarcoma (2). At present, doxorubicin, cisplatin, ifosfamide, and a high dose of methotrexate have been widely used as the preferred protocol. Different from American and European patients, however, many Asian patients fail to exhibit durable responses and succumb to these multidrug protocols. The choice of chemotherapy regimens and the choice of chemotherapy density are still controversial (3).

Multidisciplinary treatment is an essential approach for controlling the survival of patients with long-term malignant tumors (4). However, the 5-year survival rate currently ranges between 60% and 70%, reaching a plateau over the past three decades (5). Metastasis is one of the primary causes of treatment failure. Dynamically evaluating treatment response to determine the optimal therapeutic schedule and improve poor prognosis is important.

Monitoring the effect of chemotherapy on patients allows for timely adjustments of the treatment scheme (6). The serum alkaline phosphatase (ALP) level can reflect osteoblast viability. Patients with osteosarcoma have high serum ALP levels due to pathological osteogenesis (7). Its levels are significantly increased in bone cancer. The fixed-effect model suggested that higher levels of ALP may lead to a lower event-free (EF) survival rate (8). ALP may be a potential biological indicator for osteosarcoma.

Histological examination of tumor necrosis in resected specimens can also be performed to evaluate the response to neoadjuvant chemotherapy (9). Evaluation of the curative effect can indicate the risk of tumor resistance and survival. The 90% tumor necrosis threshold remains one of the most important prognostic factors in patients (10). The percentage of necrosis can only be measured in the excised lesion after the completion of neoadjuvant chemotherapy. Therefore, proposing a dynamic surrogate to predict clinical prognosis is required. This dynamic method can be used to prove the efficacy of therapeutic agents or dose adjustments in a timely manner.

Early identification of poor prognosis through imaging is crucial for osteosarcoma management (11). Previous studies have shown that volumetric changes in lesions provide better information than clinical methods (12). However, the use of the gross volume, which is susceptible to hemorrhage and edema, may lead to an increase in tumor volume. Thus, the relationship between anatomic volume, histological response of the lesion, and prognosis is still controversial. The microscopic response in malignant tumors cannot be validated using acceptable noninvasive estimations. Compared with conventional radiography and computed tomography (CT), MRI is sensitive to tumor substances and metabolism (13). Various MRI pulse sequences, such as T1-weighted spin-echo (T1) and T2-weighted fast spin-echo with fat suppression (T2), can be used to characterize different tissues (14). The influence of edema can be decreased in T1 imaging, while tissue components, such as ossification, thanatosis, and hemorrhage, can be viewed by T2. The change in the MRI signal may be indicative of tumor necrosis, and the change in osteosarcoma foci detected by MRI in the early period may be a valuable indicator for assessing the treatment effect (15). In patients with more than 90% necrosis, MRI revealed the emergence of a fat layer surrounding the tumor. We hypothesized that the presence of fat tissue is related to chemotherapy response and prognosis.

This study aimed to estimate (1) the potential of MRI to monitor the efficiency of triplet chemotherapy, and (2) the correlation between the presence of MRI after neoadjuvant chemotherapy and prognosis.

## 2 Patients and methods

### 2.1 Patients

Patients who were pathologically diagnosed with osteosarcoma between January 2009 and January 2018 were enrolled. We retrospectively reviewed all eligible patients who met the following criteria: (1) primary high-grade osteosarcoma; (2) no history of tumor treatment except biopsy; (3) availability of MRI before and after chemotherapy; and (4) chemotherapy and surgery performed at our institute. The exclusion criteria were as follows: (1) intolerance to chemotherapy; (2) active infection; or (3) metastasis on imaging. This study was approved by our Institutional Ethics Committee.

In total, 28 patients with Stage IIB osteosarcoma were treated at our hospital, including 19 male and nine female patients, with a mean age of 18.7 years (range: 6–38 years). The locations of their lesions were as follows: the proximal tibia, 12 (42.9%); proximal femur, nine (32.1%); proximal femur, three (10.7%); proximal humerus, one (3.6%); and proximal fibula, three (10.7%). All patients underwent comprehensive history-taking and a physical examination. The primary tumor size was evaluated using T1-weighted imaging. Changes in signals at T1 and T2 were used to estimate the lesion components. Chest CT or positron emission tomography (PET)-CT was used to screen for metastasis. Alkaline phosphatase (ALP) levels were measured on the 1^st^ and 5^th^ days of each course.

### 2.2 Chemotherapy and surgery

Neoadjuvant chemotherapy, consisting of 2 g/m^2^ of ifosfamide (days 1–5), 40 mg/m^2^ of adriamycin (day 5), and 8 g/m^2^ of methotrexate (day 3), was administered intravenously every 3 weeks. During the period of chemotherapy, the patients were supplied with hydration and alkalization. Magnesium isoglycyrrhizinate was used to protect the liver. Dolasetron mesilate was administered to alleviate nausea. Blood examinations were conducted to monitor marrow, kidney, and liver function every other day. The urine pH was tested regularly every day from day 3 to day 6.

All patients underwent MRI after three cycles of neoadjuvant chemotherapy. The tumor length was measured by coronal T1-weighted sequences. Edema length was measured using coronal T2-weighted sequences with fat suppression. The fat layer surrounding the tumor in the MRI was defined as the fat signal observed after chemotherapy. Two clinicians browsed every layer of the T1 images and selected the image with the maximum fat width. The image was confirmed when clinicians chose the same one. We measured the maximum fat width on transverse T1-weighted sequences.

Surgery was performed when the condition of marrow suppression was relieved (white blood cell count > 3.0 × 10^9^/L). Histological necrosis was assessed to determine the chemotherapy response following surgery. For prognostic analysis, the follow-up time was determined from diagnosis to the presence of metastasis and relapse. The follow-up time of patients without osteosarcoma development was measured on the final date.

The 3.0T limb MRI scanner included a standard T1 sequence (TR/TE 500–600/8–11 ms; slice thickness, 5 mm) and T2 sequence (TR/TE 3000–4000/15–70 ms; slice thickness, 5 mm) with fat suppression. The imaging and measurement system was a Lanyun PACS system.

### 2.3 Statistics

Serum ALP levels were measured during the hospitalization period (days 1 and 5). Logistic modeling was used to identify whether the initial length of the lesion and edema predicted changes in necrosis and survival. Differences in survival and relapse rates were analyzed using the Kaplan–Meier test. Receiver operating characteristic (ROC) curves were used to assess the sensitivity and specificity of whether the presence of a fat layer predicted the chemotherapy effect. A good chemotherapy response (> 90% tumor necrosis) was treated as a positive case. A poor response (< 90% tumor necrosis) was treated as a negative case. A logistic regression analysis was performed to determine the association between the presence of a fat layer and survival. Statistical significance was set at P < 0.05. Statistical analyses were performed using IBM SPSS Statistics for Windows, version 22.0.

## 3 Results

### 3.1 Efficiency of triplet chemotherapy

**Figure 1:**
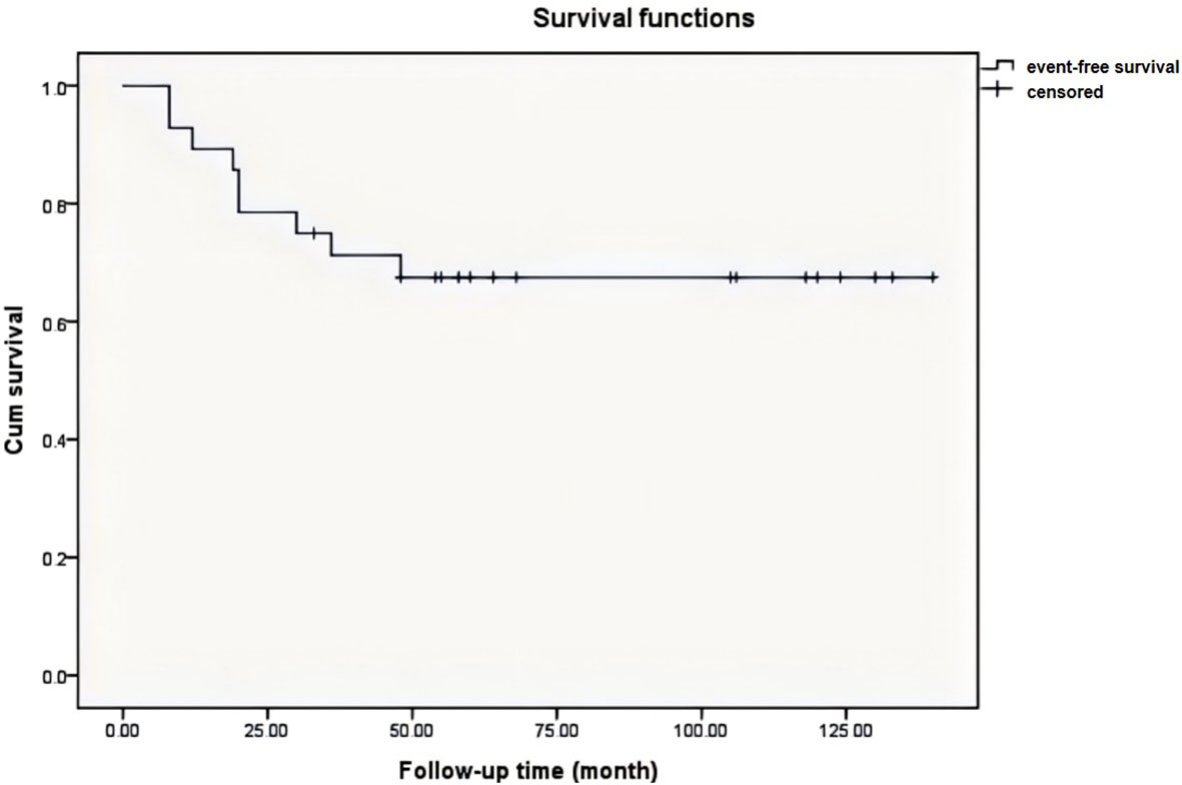
Kaplan-Meier survival curve of 28 patients shows the overall event-free survival is 67.9% at 5 years. The mean time is 64.3 ± 41.5 months in follow-up.

In our study, the mean time interval during chemotherapy was 3 months (range: 2.4 to 4 months). The median time of follow-up was 64.3 ± 41.5 months (range: 8 to 140 months). Tumor recurrence occurred in five cases (17.9%). The first evidence of tumor relapse was detected at 8 months following the treatment. Lung metastasis was first diagnosed by CT. The lung lesions, found to be gradually growing and increasing, were proved by biopsy. There were two patients with metastasis (7.1%), one of which was observed during treatment. The 3- and 5-year event-free survival rates were 75.0% and 67.9%, respectively (**Figure 1**). The mean ALP levels before and after treatment were 168.1 U/L [standard deviation (SD): 110.9] and 112.6 U/L (SD: 73.5), respectively. No increase in ALP levels was found during chemotherapy. Among the patients with relapse and metastasis, the initial ALP level was maintained at 213.8 U/L, before decreasing to 64.7 U/L following the completion of drug treatment. The independent-sample t-test identified poor significance compared to the whole sample (p = 0.22). After surgery, 17 resection samples (60%) showed > 90% necrosis, whereas six (10%) tumors showed 70% necrosis. Sensitivity was defined as necrosis > 90% (16). Linear regression analysis showed that the initial ALP and change in ALP showed a poor relationship with chemotherapy effect (p = 0.218, 0.663). ALP levels measured after chemotherapy correlated with tumor necrosis (p = 0.01).

### 3.2 Comparison of MRI characteristics and prognosis

Edema surrounding the tumor shows high signal intensity on T2, which is difficult to separate from the tumor. Due to mixing with the fatty bone marrow, the edema signal is slightly higher than that of the tumor on T1 (17). The characteristics of the MRI scans are listed in **Table 1**. After three courses of chemotherapy, the maximum dimension of the lesion on T1 was unchanged in two patients (3.5%), while enlargement was observed in three patients (10.7%). The length of edema on coronal T2 exhibited growth in two patients (7.1%). The logistic model for EF survival revealed that lesion length on initial T1 and edema on initial T2 were not effective predictors of long-term survival (**Table 1**). Change in maximum lesion length on coronal T1-weighted sequences (p = 0.044, OR = 0.035; CI: 0.01–0.911) after chemotherapy could predict EF survival. However, changes in edema on coronal T2-weighted sequences (p = 0.979, OR = 0.989, CI: 0.437–2.242), showed no relationship with prognosis. Based on tumor necrosis, the variables (extent of lesion on coronal T1-weighted sequences and extent of edema in coronal T2-weighted sequences) were considered to have poor prediction ability for chemotherapy results.

**Table 1:** Characteristics of osteosarcoma and a logistic regression analysis.

Variable	mean ± SD (cm)	odds ratios (OR)	95% confidence interval (CI)	P-value
Mean max dimension before chemotherapy (lesion on T1)	8.28 ± 3.41	1.093	0.860–1.390	0.468
Mean max dimension after chemotherapy (lesion on T1)	7.85 ± 3.40	1.144	0.895-1.461	0.282
Mean max dimension before chemotherapy (edema on T2)	9.81 ± 3.60	1.123	0.888–1.420	0.334
Mean max dimension after chemotherapy (edema on T2)	9.01 ± 3.87	1.123	0.903-1.396	0.298
The width of the fat layer	0.39 ± 0.18	0.000	0.000-0.117	0.011

### 3.3 Presence of fat layer after chemotherapy

**Figure 2:**
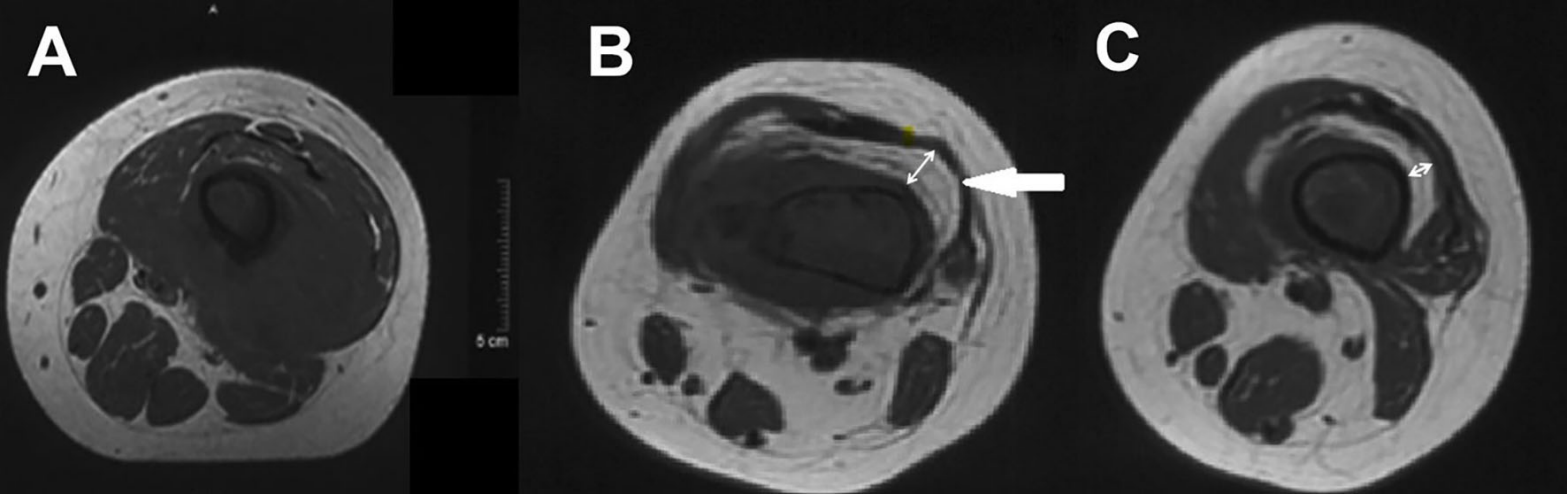
**(A)** Pre- and **(B, C)** post-chemotherapy T1-weighted cross-section MR images of a distal femur. The double directional arrow pointing towards the midpoint of the medullary cavity represents the width of fat layer.

**Figure 3:**
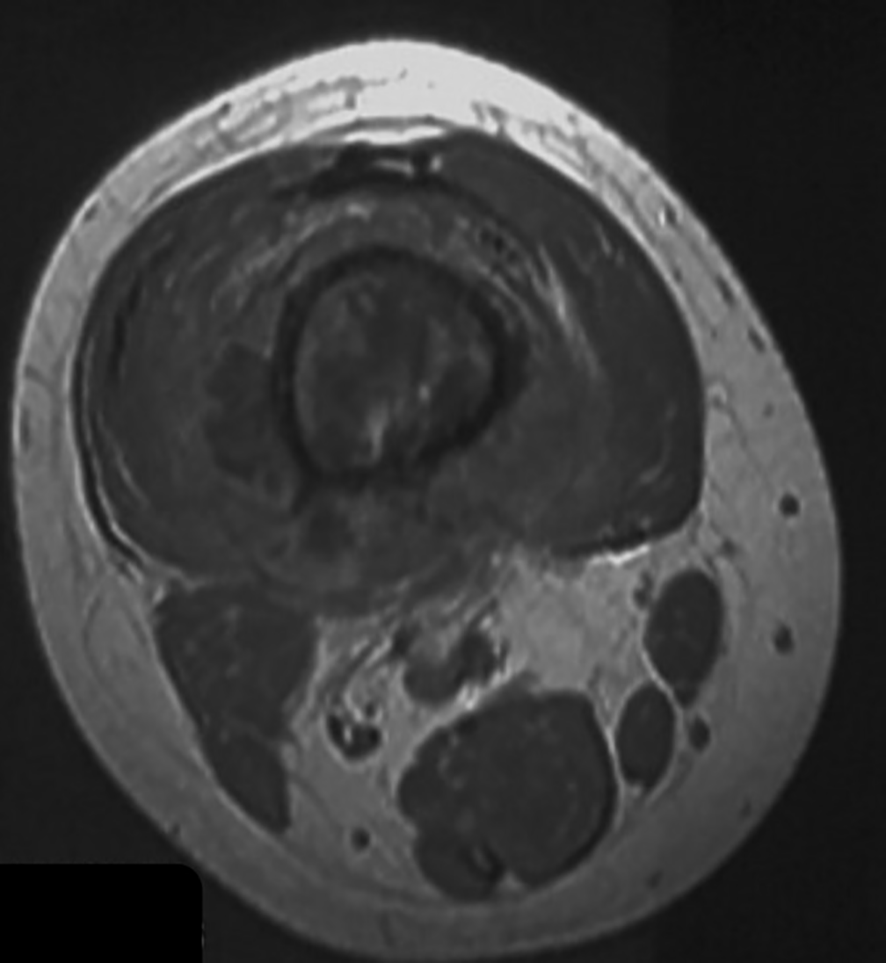
A T1-weighted image of a distal femur indicating a poor response to neoadjuvant chemotherapy.

**Figure 4:**
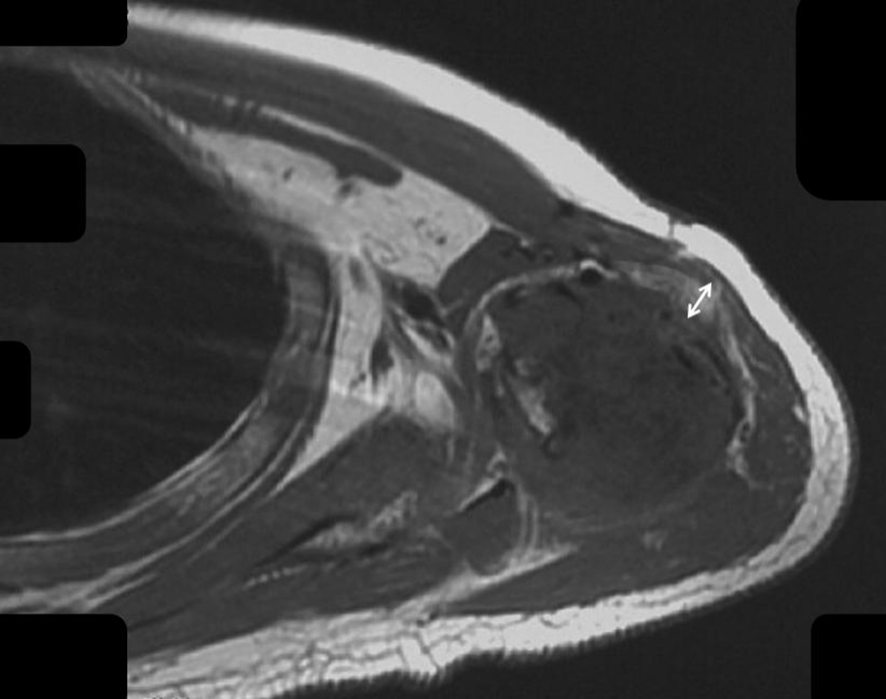
The fat layer around the humerus after chemotherapy is shown.

**Figure 5:**
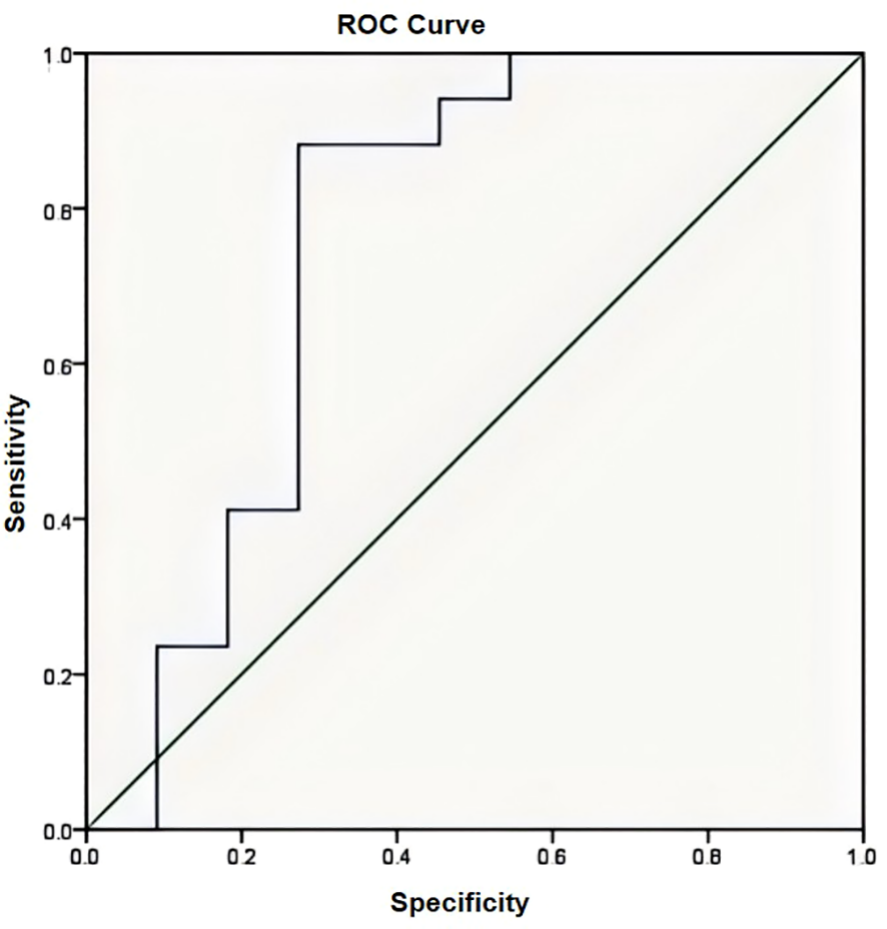
The area under the curve (AUC) was 0.759 confirmed by the ROC curve (AUC has an accuracy between 0.7 and 0.9).

Fat tissue has short T1 times and shows bright signal intensity. The maximum diameter of the fat layer on transverse T1-weighted sequences was measured as its width. The average width of the fat layer emerging after neoadjuvant chemotherapy was 0.39 cm (range: 0.06 to 0.64 cm) (**Figures 2**–**4**). The best threshold values for the fat layer were confirmed using ROC curves (**Figure 5**). The area under the curve (AUC) was between 0.5 and 1.0. The AUC for the fat layer predicting necrosis was 0.759 (CI: 54.4%–97.5%). Furthermore, at a threshold value of 0.32 cm, the sensitivity and specificity for predicting tumor necrosis were 0.882 and 0.455, respectively. Using logistic regression analysis, the significance of the fat layer as a histological indicator of necrosis was 0.03. Moreover, an increase in fat layer width was associated with a favorable prognosis (p = 0.011; OR = 0.000; CI: 0.000–0.117).

## 4 Discussion

A retrospective study determined that high ALP levels can decrease the overall survival in patients with osteosarcoma (18). A correlation between decreased ALP levels and survival was also found in patients with metastasis. However, a consensus has yet to be confirmed. We re-evaluated initial ALP levels and the fluctuation of ALP by comparing the survival rates at follow-up. Although ALP levels decreased during treatment, there was no evidence of a correlation between a decrease in ALP levels and survival. Additionally, we found no significant difference in the rate of ALP decrease among patients with disease-free status, relapse, or metastasis. However, a decrease in ALP levels after chemotherapy was found to result in a higher percentage of tumor necrosis.

The current gold standard for chemotherapy response is tumor necrosis during excision (19). However, this evaluation cannot be performed prior to surgical resection. Thus, it is difficult to detect ineffective chemotherapy in the early stages. Some patients with no metastasis and < 90% necrosis after neoadjuvant chemotherapy have favorable outcomes (20). Therefore, a more accessible factor to estimate the curative effect and prognosis is required.

Previous reports have demonstrated that tumor volume is a risk factor for event-free survival (21). The enlargement of lesions during chemotherapy may be related to active malignant tumor cells and poor drug-induced inhibition. However, some studies offer controversial perspectives, including a finding that the tumor volume before and after chemotherapy showed no reliable correlation with the efficacy of chemotherapy for osteosarcoma (22). We selected the length in the coronal plane to substitute the lesion volume. In our study, we found no significant association between maximum tumor length and prognosis. The set of length features failed to reflect the histological response, and this has several possible explanations: (i) the mineralized matrix is not affected by cytotoxic agents, (ii) hemorrhage and local necrosis, and (iii) inflammation and edema. However, changes in lesion length in coronal T1-weighted sequences showed a notable correlation with survival rate. This phenomenon suggests that tumors with good treatment response could be visibly reduced.

Multimodal MR images were extracted for consistency with the histological components. High signal intensity on T1-weighted imaging represented fat and hemorrhage. The tumor presented as an area of low signal intensity on T1. Compared to the histological appearance, a decrease in signal intensity on T2-weighted imaging with fat suppression, except for edema, was treated as tumor necrosis or calcification. Anatomical information, including the extent of intraosseous lesions, neurovascular bundles, and soft tissue involvement, is critical for the selection of surgical methods (23). MRI is commonly used to evaluate limb-saving conditions after chemotherapy. We explicitly focused on MRI signals categorized as viable or necrotic tumors to evaluate the chemotherapy response. Studies have shown that MRI can be used to detect osteosarcoma necrosis (24). However, distinguishing viable tumors from immature granulation, neovascularity, and cartilage tissues is difficult.

MRI is specific and sensitive to fat tissue. Areas with high T1 signal intensity and low signal intensity based on fat suppression correspond to areas of fat tissue. A fat layer is commonly detected around the tumor after chemotherapy. However, studies on this topic have several limitations. We assumed a feature of the fat layer to predict prognosis. The extent of the fat layer was examined to determine its association with survival. The results illustrated that different fat diameters in T1 cross sections were independent predictors of EF survival. Indeed, increases in the width of the layer predicted higher survival rates. In our study, the logistic regression analysis indicated that the fat layer was a protective factor. The presence of a fat layer after chemotherapy limits the lesion and provides a safe excision edge in the soft tissue. Separating the main artery and nerve beyond the fat layer did not increase the risk of tumor recurrence. We also compared the width of the fat layer with necrosis and determined the relationship between the fat layer and chemotherapy response. Furthermore, we observed a difference in the chemotherapy response based on the extent of the fat layer in the early phase, which was in accordance with our hypothesis. Based on this characteristic, adjusting the chemotherapy regimen over time could improve survival and reduce the risk of resistance. Seeking an optimal predictor of chemotherapy response based on the tissue components, such as the fat layer, rather than tumor volume is crucial. The signal intensity evaluated on the MRI plane tended to be more effective than those evaluated based on volume-based tumor necrosis (22). The dynamic monitoring of the tumor’s response to drugs can be implemented based on MRI. If patients with poor response are identified early, the chemotherapy regimen or dose can be changed to improve outcomes. Moreover, if the tumor remains unresponsive to drugs, surgery can be prioritized to reduce the tumor burden.

With progress in neoadjuvant chemotherapy, the survival rate of patients with osteosarcoma has improved since the 1970s (25). The Cooperative German, Austrian, and Swiss Osteosarcoma Study Group (COSS) and Rosen T-10 chemotherapy protocols are frequently used (26, 27). Three agents were administered at our institution considering the physical characteristics of the patients. Mesna was used to detoxify ifosfamide to prevent hemorrhagic cystitis. In addition, calcium folinate/leucovorin and dexrazoxane were used to detoxify methotrexate and adriamycin, respectively. We found that our chemotherapy regimen had lower toxicity and better tolerance. According to the Kaplan–Meier analysis, the EF survival rates at 3 and 5 years were 75.0% and 67.9%, respectively. The rates of relapse and metastasis did not increase.

This study has several limitations that warrant discussion. First, retrospectively collected data were limited to low-grade quality evidence. Second, the sample capacity was relatively small, and our findings need to be confirmed in a larger number of patients. Finally, tumor characteristics such as osteosarcoma subtype were not assessed. Therefore, a multicenter, prospective study with a larger sample size should be performed.

In summary, a decrease in the length of lesions was associated with good prognosis. The presence of a fat layer could predict chemosensitivity and good prognosis in patients with osteosarcoma. Validation of these findings in future studies will help to avoid resistance without surgery, which may lead to a better prognosis.

## Data Availability

The raw data supporting the conclusions of this article will be made available by the authors, without undue reservation.
